# Acquired White Oral Lesions with Specific Patterns: Oral Lichen Planus and Lupus Erythematosus

**DOI:** 10.5826/dpc.1103a74

**Published:** 2021-05-20

**Authors:** Marco Manfredini, Gioia Pedroni, Laura Bigi, Roberto Apponi, Alberto Murri dello Diago, Annunziata Dattola, Francesca Farnetani, Giovanni Pellacani

**Affiliations:** 1 Dermatology Unit, Department of Surgical, Medical, Dental & Morphological Sciences with Interest in Transplant, Oncological & Regenerative Medicine, University of Modena & Reggio Emilia, Modena, Italy; 2 Dental Unit, Department of Surgical, Medical, Dental & Morphological Sciences with Interest in Transplant, Oncological & Regenerative Medicine, University of Modena & Reggio Emilia, Modena, Italy; 3 Dermatology Clinic, Department of Systems Medicine, Tor Vergata University, Rome, Italy; 4 Dermatology Clinic, Department of Clinical, Internal, Anesthesiological and Cardiovascular Sciences, Sapienza University of Rome, Rome, Italy

**Keywords:** Oral white lesions, lichen planus, lupus erythematosus, mucoscopy

## Abstract

**Background:**

Diagnosis of oral white lesions might be challenging. These lesions represent a wide spectrum of diseases with different etiology and prognosis. Oral white lesions can be categorized into two major groups, congenital and acquired, according to their development, and in four subgroups: lesions which can be scraped off or not and lesions with special pattern or not.

**Objectives:**

The aim of this manuscript is to review, from diagnosis to treatment, the current knowledge on oral white lesions with specific pattern.

**Methods:**

A review on oral white lesions with specific pattern was conducted on PubMed and Scopus from inception to January 2021.

**Results:**

Among acquired lesions with specific pattern two clinical entities are mostly represented: Oral lichenoid reactions and Lupus erythematosus. The etiology of both diseases is still not known but their pathogenesis is mainly immunological. At present the mucoscopic features of those disease have been described only in few case reports or case series. Immunomodulatory therapies are often the agents of choice for their treatment.

**Conclusions:**

The collaboration of dermatologists and dentists as a team is important for early diagnoses and effective treatments. Mucoscopy is a promising technique which may reveal important features for the differentiation of OLP and LE oral white lesions.

## Introduction

The diagnosis of white oral lesions can be challenging. These lesions represent a wide spectrum of diseases that vary in etiology and prognosis [1]. The diagnosis is complex because many lesions of the oral mucosa are autoimmune in nature or are the result of immunologically mediated diseases such as lichen planus (LP), bullous diseases, pemphigus vulgaris, mucous membrane pemphigoid, recurrent aphthous stomatitis, and erythema multiforme [2–5]. Numerous familial tumor syndromes also have distinctive oral mucosal findings that may facilitate an early diagnosis [6]. Often, the dermatologist and dentist work as a team to make early diagnoses and provide effective treatments. Indeed, many of these pathologies are of concern to multiple medical disciplines [7,8].

According to a recent paper, white oral lesions can be categorized according to their nature of development into two major groups, namely congenital and acquired, and four subgroups: lesions which can be scraped off or not, and lesions with special patterns or not [1]. Clinical features of white lesions, such as papular, anular, reticular or erosive-ulcerative patterns, or a combination of them can be used to differentiate white patterned lesions from non-patterned ones [1]. There are 2 types of acquired lesions with specific patterns: oral lichenoid reactions and lupus erythematosus (LE). The first group includes: oral LP; oral LP-associated with underlying diseases (eg, thyroid disease, dyslipidemia, diabetes mellitus, hepatitis C virus infection); lichenoid contact reaction (LCR); drug-induced lichenoid reactions (DILR); and graft-versus-host reaction (GVHD). This article reviews current knowledge on white oral lesions with specific patterns, from diagnosis to treatment.

## Methods

Bibliographic research for articles on the clinical features and treatment strategies of oral lesions of oral LP and LE was conducted on PubMed and Scopus databases from their inception to January 2021. We also searched for the mucoscopic pattern of oral LP and LE using these search strings: (“dermoscopy” AND “oral” AND “white”), (“dermoscopy” AND “mucosal”), (“mucoscopy” AND “lichen”), (“mucoscopy” AND “lupus”), (“mucoscopy” AND “white”). Articles reporting studies on pigmented lesions or lesions of the genital area were excluded.

## Oral LP

**Figure 1:**
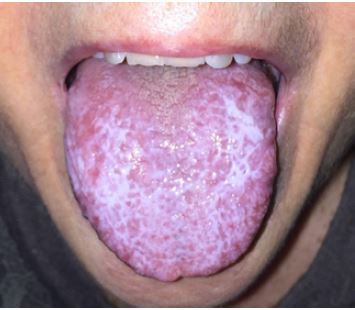
Oral lichen planus affecting the tongue. The lesion is characterized by the presence of evident white reticular lines (Wickham striae).

Oral LP is the most frequent disease of the white oral lesion group [9]. It affects 1%–2% of the population. It is associated with skin lesions in 60%–70% of cases, and is the only manifestation in 15%–25% of patients. A typical manifestation of OLP are multiple, symmetrical lesions that appear in keratotic/papular, erythematous/erosive or vesicle-bullous form [9]. Generally, the lesions are predominant within the lips, in the buccal mucosa and on the dorsal tongue [9,10]. The leukokeratosis form, showing a distinctive network of white lines (Wickham striae), is the most frequent, while the bullous form is rare (Figure 1). The erosive form is characterized by areas, more or less extensive, of ulcerative mucosa.

Although the exact pathogenesis of OLP is still unknown, immunological mechanisms are likely to have an important role. OLP is considered a T cell mediated immune (autoimmune) disease in which CD8+T-cells trigger apoptosis of basal epithelial cells [9,11,12].

It is still debated if oral LP is associated with other systemic comorbidities such as diabetes mellitus (DM), thyroid disease and chronic liver disease, especially HCV infection. These associations have been observed in several studies, with an incidence ranging from 0.5% to 35% in oral LP patients [13–15]. OLP lesions often have a persistent course and propensity for malignant transformation over time [16]. Oral squamous cell carcinoma (OSCC) is often characterized by an insidious onset, difficult diagnosis, fast evolution with frequent metastasis and disfiguring surgical treatments. A histopathologic analysis of the lesion obtained through scalpel or punch biopsy should be performed without delay if OSCC is suspected [17–19].

To our best knowledge, only 5 case reports have been published on the mucoscopic appearance of oral LP. These reports have described oral LP as being characterized by white reticular lines over an erythematous-violaceous background with curved vessels. Superficial crusting or scaling blunted papillae, tiny erosions, interspersed clods and a polymorphic vascular pattern have been reported [20,21].

### Oral LP Treatment

Both topical and systemic corticosteroids are used to treat oral LP. Fluocinonide embedded in an adhesive gel has been used with good results for both the leukokeratotic and erosive forms. The therapy lasts about 9 weeks without side effects. Other topical corticosteroids used include: 0.1% triamcinolone acetonide, 0.1% fluocinolone acetonide, 0.05% fluocinolone acetonide, and 0.05% clobetasol propionate [1]. Betamethasone has also been used with good results. In the most severe forms, systemic corticosteroids may be used, usually at the same dosage employed in cutaneous LP. Although different dose regimens have been proposed, the minimal effective daily dose of prednisone is usually 15–20 mg for 2–6 weeks [1].

When both topical and systemic corticosteroids are not sufficient, immunomodulatory agents, such as calcineurin inhibitors (CI) may be used. Cyclosporin has been prescribed both in adhesive bases and as a mouthwash, even though it is not always effective [22]. Topical tacrolimus is a more potent CI which can be safely used as a valid treatment alternativo of recalcitrant and erosive OLP. Pimecrolimus topic cream has been also successfully prescribed for the treatment of erosive OLP lesions.

Treatment with efalizumab, a recombinant humanized monoclonal immunoglobulin G antibody, led to the improvement of oral lesions present on buccal mucosa and tongue with an initial dose of 0.7 mg/kg, followed by a dosage of 1.0 mg/kg per week [23]. Efalizumab inhibits the binding of leukocyte function antigen-1 (LFA1) to the intercellular adhesion molecules-1 (ICAM-1), thereby inhibiting the adhesion of leukocytes to other cell types lead to the improvement of OLP via decreased activation and trafficking of T lymphocytes, which play a vital role in its pathologic development of OLP.

Mycophenolate mofetil showed to be effective long term in severe cases of OLP [24]. Good results, both in erosive and in atrophic OLP, are obtained with topical tretinoin 0.1%, though relapses are frequent. The same frequency of relapse occurs with isotretinoin gel; this, however, reduces the clinical manifestation and symptoms in 80% of patients. Retinoids are generally less effective compared with topical corticosteroids [25].

Several other drugs have been used for treatment of mucosal LP: griseofulvin, dapsone, hydroxichloroquine, thalidomide, levamisole. Long-term (3 to 6 months) administration of griseofulvin was shown to result in complete improvement in 86% of patients with LP. In particular oral erosive lesions have responded favorably to this drug. A complete response of disease, including oral lesions was observed in patients treated with metronidazole, 500 mg twice daily for 20 to 60 days [26].

Thalidomide in dose range of 50–150 mg daily produced a regression of OLP. Daily or prolonged treatment periods at higher doses (300 mg) may be required to prevent recurrences [12].

Treatment with Levamisole plus Vit B12 resulted in clinical in oral signs and symptoms of OLP. Moreover, the treatment reduced high serum anti-gastric parietal cell autoantibody (GPCA) level (a potential marker of OLP) to undetectable level [27]. Oral Apremilast has been used for severe erosive OLP at dosage of 30 mg twice a day; following completion of 12 weeks treatment, marked improvement was observed in buccal and gingival lesions, with a significant reduction in pain and discomfort [28].

Recently, a high expression of human beta-defensin 2 (hBD-2), a potent antimicrobial peptide, has been reported in OLP lesions. suggesting that it may be harnessed for therapeutic interventions in OLP [29]. Photodynamic therapy (PDT) has been successfully used in severe refractory cases of erosive OLP [10,11]. However, a systematic review on the efficacy of photodynamic therapy (PDT) in the management of symptomatic OLP reported inconsistent results. On the other hand, a randomized clinical trial indicated a better efficacy of PDT therapy compared to corticosteroids. Generally, PDT treatment was able to reduce pain and burning sensation and to decrease the size of the lesions in symptomatic OLP patients. Several studies reported the effects of laser therapy on the erosive OLP, including the use of 980-nm diode laser, carbon dioxide laser evaporation, bio stimulation with a pulse diode laser using 904-nm infrared rays, and low dose excimer 308-nm laser with ultraviolet (UV) B rays. Although promising results were reported by some studies, the effectiveness of laser therapy in OLP is yet to be proven [30]. Among non-pharmacological strategies, the use of ozone (O3) as a complementary medical approach has increased progressively. Ozone is a highly unstable atmospheric gas that rapidly decays into normal oxygen(O2). Although not being a radical molecule, O3 is a very strong oxidant and, due to this highly toxic property, it has been widely used as a disinfectant and germicidal agent, also for medical purposes. In addition, ozone administration as a mixture of O2-O3 gases has proven to improve metabolic activity and to exert therapeutic effects in numerous diseases. The use of ozonized water in association with conventional topical corticosteroids application in OLP resulted in a significant improvement of sign and pain [31].

### Lupus erythematosus

Lupus erythematosus (LE) is an autoimmune disease that can be classified into three distinct forms: systemic lupus erythematosus (SLE), subacute cutaneous lupus erythematosus (SCLE) and chronic cutaneous lupus erythematosus (CCLE) [32,33]. Skin lesions (85%) include the characteristic butterfly rash (40%–50%), alopecia, photosensitivity, Raynaud’s phenomenon, livedo reticularis, urticaria, erythema, telangiectasias, and cutaneous vasculitis [33–36]. Sunlight often aggravates the malar rash [34,35]. The etiology remains unknown, however increased autoantibody generation with the imbalanced function of T lymphocytes have been reported. There is an extensive range of clinical symptoms for SLE, characterized by a remarkable clinical heterogeneity due to synchronous and non-synchronous involvement of several organs with variable severity [34,35].

Oral manifestations of LE (9%–45% in SLE, 3%–20% in CCLE) include ulcerations, erythematous lesions, hyperkeratosis, honeycomb plaques, and discoid lesions. Lesions generally affect the palate, buccal mucosa, and gingivae. Sometimes, the vermilion zone of the lower lip (lupus cheilitis) is also affected [37]. Ulcers are often aphthous-like with a white to yellow coating and a peripheral red rim especially in the hard palate [38]. A honeycomb plaque is a rare condition, revealed as a chronic, well-defined plaque along with white lacy hyperkeratosis and buccal erythema [38]. Lesions generally affect both lining and masticatory mucosa, however they are less hyperkeratotic on the lining mucosa (eg, soft palate). Discoid oral lesions appear as whitish striae generally radiating from the central erythematous area (“brush border” pattern), which makes it difficult to distinguish them from oral candidiasis or OLP if there are no systemic or cutaneous findings. Lupus cheilitis is an inflammatory condition of the lips presenting as a small or diffuse, erythematous and edematous lesion that might develop into crusty painful ulcers. This condition usually affects the vermilion zone of the lower lip [37–39]. Oral manifestations of CCLE are similar to erosive OLP with an ulcerated or atrophic, erythematous central area and peripheral white, fine, radiating striae. Occasionally the central region shows a fine stippling of white dots along with erythema. However, the oral features are generally accompanied with skin lesions. When ulcerative and atrophic oral lesions come in contact with acidic or salty foods, a pain similar to erosive OLP, might be experienced. Oral features of SCLE are the same as those of CCLE [38]. Diagnosis of SLE can be difficult in the early stages because of its polymorphic clinical course usually characterized by remission and flares. American Rheumatism Association has defined several clinical and laboratory criteria for the diagnosis of SLE [38]. Occasionally, oral lesions characterized by the presence of radiating white striae resembling Wickham’s striae, has been reported, therefore, biopsy is required for definite diagnosis [32,38].

To our knowledge, only 3 case series on the mucoscopic appearance of oral and lips LE lesions have been reported, characterized by white halos and network-like white lines with long linear and dotted vessels over a diffuse erythematous background [20,40,41].

### Treatment

At present, therapy is based on combinations of antimalarials (mainly Hydroxichloroquine or Quinacrine), considered as the backbone of LE treatment, glucocorticoids, and immunosuppressive drugs [33].

Effective protection from ultraviolet exposure with broad-spectrum sunscreens and smoking cessation are highly recommended. In addition, vitamin D supplementation is suggested in all patients with low vitamin D levels [35].

Topical anti-inflammatory agents are the treatment of choice for oral ulcers (eg, 0.1% triamcinolone oral paste) shortening the course and severity of the oral lesions [42]. The duration of corticosteroid usage depends on the severity of the symptoms. If the oral lesions are refractory to the treatment, then more potent (eg, betamethasone or clobetasol in oral preparation) or systemic drugs may be needed. Steroid-sparing agents, such as calcineurin inhibitors (eg, 0.03% or 0.1% tacrolimus) are also applicable when the side effects of corticosteroids pose some concern [42]. The alternative route of corticosteroid administration, intra-lesional injection, is rarely used due to pain.

Mild LE cases can be successfully managed by means of NSAIDs along with anti-malarial agents. Systemic corticosteroids in combination with other immunosuppressive agents and immunomodulators are frequently used for more severe conditions [33,38]. Despite limited randomized evidence, immunosuppressive agents such as Methotrexate, Azathioprine, and Cyclosporine are considered in SLE patients who respond inadequately to antimalarials and glucocorticoids. Other agents include retinoids, dapsone, mycophenolate mofetil or EC-mycophenolic acid and Thalidomide [38].

Among biological therapies, B-cell-targeted therapy showed the most promising results. Several B cells targeting therapies including targeting of BCR signaling and B cell depletion are reported up to date [38,43]. Belimumab and Rituximab have shown efficacy in mucocutaneous manifestations of SLE. According to the EULAR recommendations for the management of systemic lupus erythematosus Belimumab should be considered in extrarenal disease with inadequate control to first-line treatments, and inability to taper glucocorticoid daily dose to acceptable levels [38]. Rituximab (RTX) is currently only used off-label, in patients with severe renal or extrarenal (mainly haematological and neuropsychiatric) disease refractory to other immunosuppressive agents and/or belimumab, or in patients with contraindications to these drugs.

Other therapies involving the targeting of T cells and cytokines are still under investigation [44]. Several molecules for SLE treatment are currently at advanced stages of research trial (Phase III and IV studies) and may provide novel therapeutic strategies for SLE: Tabalumab and Blisibimod that are binding B-cell activating factor (BAFF), Dapirolizumab pegol, an anti-CD40L Fab’ fragment, Anifrolumab, a human monoclonal antibody to type I interferon receptor subunit [45], and the dual-target biological agents telitacicept, which is a novel recombinant TACI-Fc fusion protein able to inhibit BAFF and A proliferation inducing ligand (APRIL) cytokines at the same time [43,44].

## Conclusions

A team collaboration between dermatologists and dentists is important to allow early diagnoses and effective treatments of mucosal lesions of the oral cavity. OLP and LE oral lesions have been recently defined as “acquired oral white lesions with specific pattern” because they present as whitish lesions of the oral cavity, characterized by similar mucoscopic features such as the presence of white structures, network-like white lines and erosion/ulceration. Both diseases can affect the lips and the buccal mucosa. OLP have been frequently reported on the dorsal tongue, while LE was more frequently reported on the palate. Further studies are needed to better characterize and differentiate OLP and LE oral white lesions.
